# Do differences in the administrative structure of populations confound comparisons of geographic health inequalities?

**DOI:** 10.1186/1471-2288-10-74

**Published:** 2010-08-18

**Authors:** Andrew L Jackson, Carolyn A Davies, Alastair H Leyland

**Affiliations:** 1 MRC Social and Public Health Sciences Unit, 4 Lilybank Gardens, Glasgow, UK, G12 8RZ

## Abstract

**Background:**

Geographical health inequalities are naturally described by the variation in health outcomes between areas (e.g. mortality rates). However, comparisons made between countries are hampered by our lack of understanding of the effect of the size of administrative units, and in particular the modifiable areal unit problem. Our objective was to assess how differences in geographic and administrative units used for disseminating data affect the description of health inequalities.

**Methods:**

Retrospective study of standard populations and deaths aggregated by administrative regions within 20 European countries, 1990-1991. Estimated populations and deaths in males aged 0-64 were in 5 year age bands. Poisson multilevel modelling was conducted of deaths as standardised mortality ratios. The variation between regions within countries was tested for relationships with the mean region population size and the unequal distribution of populations within each country measured using Gini coefficients.

**Results:**

There is evidence that countries whose regions vary more in population size show greater variation and hence greater apparent inequalities in mortality counts. The Gini coefficient, measuring inequalities in population size, ranged from 0.1 to 0.5 between countries; an increase of 0.1 was accompanied by a 12-14% increase in the standard deviation of the mortality rates between regions within a country.

**Conclusions:**

Apparently differing health inequalities between two countries may be due to differences in geographical structure *per se*, rather than having any underlying epidemiological cause. Inequalities may be inherently greater in countries whose regions are more unequally populated.

## Background

Inequalities in health exist at many levels: between individuals, neighbourhoods, socio-economic groups, regions, countries and entire continents. Attempts to reduce social inequalities in health are often focused on geographical disparities since policy is most easily directed at administrative units such as local government [1,2]. Geographic clusters of people can also be used as a proxy for underlying socio-economic or genetic factors since individuals nearby in space and time may be more similar than individuals separated by large distances [3]. Accurate monitoring and description of geographic health inequalities is essential if we are successfully to reduce them. Moreover, the ability to develop hypotheses regarding the social, cultural, behavioural, political or health care differences between countries means that studies comparing inter-country variability in the magnitude of inequalities in health are of key importance in identifying opportunities to achieve these reductions [4]. Modelling the variance may be a means of gaining insight into health inequalities and developing hypotheses regarding contexts [5].

By exploiting the inherent hierarchical structures present in populations (people live within neighbourhoods which are nested in regions nested in countries), multilevel models provide an appropriate statistical method for describing and explaining geographic health inequalities on a range of spatial scales [6,7]. Variation in health statistics between geographic units derived from variance components models can be used as a direct measure of inequalities. Larger variance or standard deviation implies greater variation as the difference between fixed quantiles, e.g. the 5^th ^and 95^th ^centiles is greater. Patterns or trends of health inequalities can be assessed by comparing this variation across countries, or within a country over time [8].

However, one may rightly question whether it is fair to make direct comparisons of health inequalities between countries with different internal administrative unit sizes. When analyzing health data which have been aggregated into pre-defined spatial units we are likely to experience the modifiable areal unit problem (MAUP), whereby statistical bias or variation can occur due to the arbitrary nature of the aggregation of individuals into areas [9-11]. More specifically the MAUP consists of two interrelated components; the scale effect whereby statistical bias can occur when the information is grouped at different levels of spatial resolution i.e. the bias occurs due to the differing number of areas used in the analysis; and the zoning effect whereby bias is a result of the various ways areas can be aggregated at a given scale, and is not due to the variation in area size. Routinely collected data, which are frequently used in public health, are often restricted by the boundaries of the units for which the data have been provided. Such boundaries, in for example census data, are generally not designed to delineate communities or reflect homogeneity in terms of health. Further, although these boundaries are often designed to meet constraints on population thresholds, there still remains much variation, in terms of population size, between such areas and therefore when comparing health outcomes such as mortality rates, or inequalities in mortality rates, across regions we are faced with a variation of the MAUP scale effect and must seek potential solutions.

In this paper we examine whether spatial inequalities in mortality between countries are associated with the distribution in regional population sizes and in so doing potentially provide a statistical method of adjusting for the variation in administrative unit sizes within countries. Addressing this issue will also provide clues as to whether it is fair to compare health inequalities over time when existing boundaries are changed within a country (such as during Local Government reorganisation in Great Britain 1995-1998). For example, historical differences in the formation of administrative regions between regions or countries may exacerbate or occlude existing health inequalities, while the apparent increase or decrease in inequalities over time may in part be due to geographic boundary changes rather than any underlying epidemiological reasons [12]. This latter point has been recognised as a potential confounding factor, especially in the UK, where methods for creating consistent boundaries or adjusting denominator populations have been developed to tackle this specifically [13,14].

Furthermore, region boundary definitions can be made for several reasons [15], many of which may be at odds with statistical goals of grouping common ecological factors [16] - this is certainly true for electoral areas in the UK [17]. Although we do not directly examine the issue of boundary changes over time and their relationship with health inequalities, exploring what effect differing population structures at one time point have on such health inequalities will provide clues to both situations where, essentially, we are interested in answering the same question - does comparing differently structured administrative units influence our interpretation of health inequalities?

We use a European dataset of 20 countries comprising administrative areas mainly at the NUTS II level or an equivalent level below the country level (e.g. Regions in France, Counties in the UK etc.) to explore whether differences in the structure of geographic units used for reporting data affect regional inequalities in mortality rates. Despite mortality and population being measured at the standard NUTS II level in our data, the number of regions and the distribution in region population size within countries varies across Europe. As discussed, the MAUP, and in particular the scale effect, needs to be addressed as the differing levels of spatial resolution are likely to be influencing our interpretation of within-country health inequalities. Assuming that countries with larger variation in mortality rates between their internal regions indicate greater inequalities than countries with smaller variation, we model this variation by relating it to the distribution of region populations within each country. This is logical as it is likely that geographical regions with larger populations are more diverse in, for example, socio-economic or cultural characteristics than areas with smaller populations.

Our hypothesis can be summarised as follows: countries with larger variation between mortality rates in their internal regions have greater geographic inequalities in this health measure than those with lower variation; and we expect this variation to be related to the distribution of internal region sizes as ecological determinants of health will be more strongly clustered in small regions than large ones. We extend a basic variance components model and explicitly model the variance to describe how inter-regional variation (within a given country) relates to the mean region population size and to inequality in region population size of that country.

## Methods

Our dataset contains 20 European countries, each of which comprises their respective administrative regions below national level (most of these were NUTS II units at the time or were an equivalent level below the country level: Table 1) [18]. The data represent the number of deaths from all causes per region for males under 65 years between the mid-points of 1990 and 1991 [19]. The data were originally divided into 14 age groups (0, 1-4, 5-9,... 60-64). We removed this sub-division in our analyses by calculating the observed and predicted number of deaths per region following the calculations used to generate indirectly Standardised Mortality Ratios [20] (SMRs). The reference population used for this standardisation was the complete 20 country dataset in our study.

**Table 1:** Number of administrative regions, mean, minimum and maximum region population size and Gini coefficient for each country

Country	Number of regions	Mean Region Population	Minimum Population *Region*	Maximum Population *Region*	Gini Coefficient	***σ***_***u(j)***_**(model 1)**
Austria	9	388511	116581 *Burgenland*	630930 *Lower Austria*	0.30	0.18
Bulgaria	9	441315	260422 *Mikhaylovgrad*	544456 *Plovdiv*	0.10	0.05
Czech Rep.	8	590147	309375 *Jihoèeský*	894204 *Jihomoravský*	0.19	0.11
Denmark	15	154584	19057 *Bornholm*	262859 *Århus*	0.25	0.14
Finland	12	190464	10549 *Ahvenanmaa*	549665 *Uusimaa*	0.39	0.12
France	22	1171828	106513 *Corsica*	4796173 *Île de France*	0.37	0.11
Germany	16	2255913	286632 *Bremen*	7519917 *North Rhine-Westphalia*	0.44	0.18
Greece	13	354070	79930 *Ionian Islands*	1475350 *Attica*	0.46	0.13
Hungary	20	232590	96365 *Nόgrád*	821739 *Budapest*	0.29	0.09
Italy	20	1275911	50272 *Valle d'Aosta*	3803059 *Lombardy*	0.43	0.09
Netherlands	12	579653	105133 *Flevoland*	1429211 *Zuid-Holland*	0.40	0.05
Norway	18	106327	34522 *Finnmark*	373730 *Oslo og Akershus*	0.32	0.12
Poland	49	363065	109241 *Chelm*	1855334 *Katowice*	0.31	0.09
Romania	41	264735	107245 *Covasna*	1006544 *Bucharest*	0.24	0.09
Russian Fed.	79	850337	80665 *Chukotka*	3686473 *Moscow (city)*	0.39	0.10
Spain	18	988100	57000 *Ceutay Melilla*	3088000 *Andalusia*	0.47	0.12
Sweden	24	160028	24045 *Gotland*	709982 *Stockholm*	0.34	0.09
Switzerland	26	117639	6046 *Appenzell-Inner Rhoden*	499548 *Zürich*	0.51	0.08
UK	56	462425	48617 *Isle of Wight*	2970235 *Greater London*	0.42	0.13
Ukraine	26	878529	399913 *Chernivtsi*	2293997 *Donetsk*	0.25	0.08

Summary of the 20 countries detailing the number of administrative regions, the mean, minimum and maximum region population size and the Gini coefficient describing inequality in region size. Population values refer to males aged 0-64 between the mid-points of 1990 and 1991. Fitted values describing the inter-regional variation at country-level (*σ*_*u(j)*_) derived from the simple random relationship model 1 are given.

**Figure 1:**
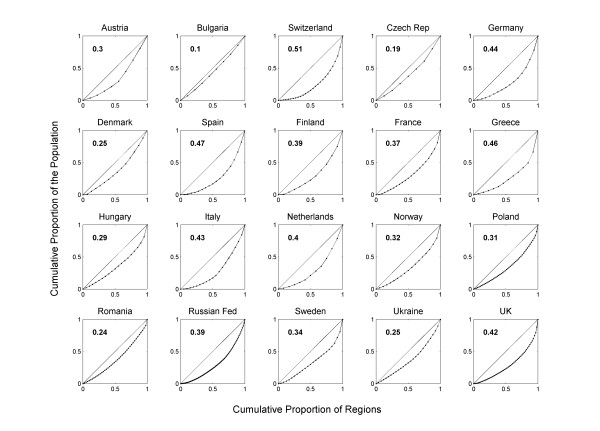
**Lorenz curves illustrating inequalities in region population size within twenty countries**. Each panel shows the cumulative proportion of the population against the cumulative proportion of regions (solid line & filled circles). The corresponding Gini coefficient is given in the top-left of each panel. The line of perfect equality (1:1) is included (solid line).

We consider two measures of the differences in population structure between countries and consider whether each is related to the variation in mortality rates within countries. Firstly, we use the mean region size, which varies substantially between countries, as a descriptor of internal region size structure. Furthermore, some countries show near uniformity in the size of regions whilst others comprise some small and some large regions. We therefore use a Gini coefficient to describe population size inequalities between regions. The Gini coefficient is generally used to express inequalities in income or mortality itself between populations although it can be applied widely to describe any inequality [21,22]. For a country such as Austria which comprises 9 regions, each region would contain 1/9 of the total population if all the regions were equal in population. However, some of Austria's regions contain much less than a ninth of the population and others much more. By plotting the (ranked) cumulative proportion of the population in the regions against the cumulative proportion of regions (in the case of Austria this is [1/9, 2/9, 3/9, ... 9/9]) we obtain Lorenz curves for each country (Figure 1). We used Brown's [23] method to calculate the Gini coefficient defined as twice the area between the Lorenz curve and the diagonal line describing perfect equality (1:1) separately for each country. Low Gini coefficients indicate countries with roughly equally sized regions, those with larger indices have greater inequality in the distribution of the population among the administrative regions.

Multilevel models were used as they allow recognition of the natural geographical hierarchy in the data (counts of death within regions are nested within countries) by partitioning the residual variance into a within-country component (the variance of the region-level residuals) and a between-country component (the variance of the country-level residuals) [6,7]. One must use this approach to take into account the correlation that exists between mortality rates in regions within the same country; ignoring this, and assuming independence between such observations, will lead to an underestimation of standard errors. This method has the added benefit of allowing us to relate descriptors of within-country region size directly to the between-region variation (i.e. geographical inequalities) in mortality rates. The observed numbers of deaths per region (*O*_*ij*_) were fitted to a Poisson distribution with rate parameter *μ*_*ij *_for the *i*th region in the *j*th country such that

(1) $$O_{ij}\sim Poisson\left(\mu_{ij}\right)$$

(2) $$\log\left(\mu_{ij}\right)=\log\left(E_{ij}\right)+\beta_{0}+v_{j}+u_{ij}$$

where *E*_*ij *_is the expected number of deaths in the *ij*th region (and its logarithm is used as an offset in the estimation of *μ*_*i*_). Using the estimated number of deaths in this manner is equivalent to modelling indirectly standardised mortality rates, hence we are effectively modelling the proportional excess or deficit mortalities compared with the expected number [6]. The country level residuals ν_*j *_are assumed to be normally distributed with mean zero and variance $\sigma_{v}^{2}$; the variance of the region level residuals, $\sigma_{u\left(j\right)}^{2}$, is allowed to vary between countries:

(3) $$u_{ij}\sim N\left(0,\sigma_{u\left(j\right)}^{2}\right)$$

(4) $$v_{j}\sim N\left(0,\sigma_{v}^{2}\right)$$

We were interested in exploring the variation between regions within a country as functions of that country's Gini coefficient (*G*_*j*_) and mean region size (*R*_*j*_). This was achieved by modelling within country standard deviation (*σ*_*u*(*j*)_) using a variety of relationships detailed in Table 2. Model 1 is a simple random relationship and acts as a null model against which we can compare linear relationships between either *σ*_*u*(*j*) _or log(*σ*_*u*(*j*)_) and the Gini coefficient and/or mean region size. Note that we have run versions of most models both with linear effects with random terms (e.g. model 2 where an additional standard deviation *σ*_*r *_is included for the residuals in the regression) and with (nearly-) deterministic linear effects (e.g. model 3).

**Table 2:** Summary of the Gini coefficient and mean region size models and results

			Parameter Estimates				
				
Model #	Description	Equation for within country variation	*β*_0_	*β*_1_	*β*_2 _(95% CrI)	*β*_3 _(95% CrI)	DIC
*Baseline random model*							
1	Random	*σ*_*u*(*j*) _~ *U*(0,1)	-0.17	-	-	-	5605
*Gini coefficient models*							
2	Log	$\log\left(\sigma_{u\left(j\right)}\right)\sim N\left(\beta_{1}+\beta_{2}G_{j},\sigma_{r}^{2}\right)$	-0.18*	-2.71*	1.14 (-0.2 2.37)	-	5601
3	Log (deterministic)	log (*σ*_*u*(*j*)_) ~ *N*(*β*_1 _+ *β*_2_*G*_*j*_, 0.0001)	-0.19*	-2.74*	1.28* (0.44 2.11)	-	5600
4	Linear	$\sigma_{u\left(j\right)}\sim N\left(\beta_{1}+\beta_{2}G_{j},\sigma_{r}^{2}\right)$, *σ*_*u(j) *_> 0.0001	-0.18*	0.06*	0.11 (-0.02 0.22)	-	5601
5	Linear (deterministic)	*σ*_*u*(*j*) _~ *N*(*β*_1 _+ *β*_2_*G*_*j*_, 0.0001), *σ*_*u(j) *_> 0.0001	-0.19*	0.06*	0.12* (0.02 0.22)	-	5600
*Mean region size models*						(Million inhabitants)	
6	Log	$\log\left(\sigma_{u\left(j\right)}\right)\sim N\left(\beta_{1}+\beta_{3}R_{j},\sigma_{r}^{2}\right)$	-0.18*	-2.43*	-	0.19 (-0.04 0.42)	5601
7	Log (deterministic)	log (*σ*_*u*(*j*)_) ~ *N*(*β*_1 _+ *β*_3_*R*_*j*_, 0.0001)	-0.18	-2.40*	-	0.18* (0.05 0.32)	5601
8	Linear	$\sigma_{u\left(j\right)}\sim N\left(\beta_{1}+\beta_{3}R_{j},\sigma_{r}^{2}\right)$, *σ*_*u(j) *_> 0.0001	-0.14	0.09*	-	0.02 (-0.00 0.05)	5602
9	Linear (deterministic)	*σ*_*u*(*j*) _~ *N*(*β*_1 _+ *β*_3_*R*_*j*_, 0.0001), *σ*_*u(j) *_> 0.0001	-0.22*	0.09*	-	0.02* (0.00 0.04)	5600
*Combined Gini coefficient and mean region size models*						(Million inhabitants)	
10	Log	$\log\left(\sigma_{u\left(j\right)}\right)\sim N\left(\beta_{1}+\beta_{2}G_{j}+\beta_{3}R_{j},\sigma_{r}^{2}\right)$	-0.19	-2.70*	0.85 (-0.57 2.17)	0.14 (-0.10 0.38)	5601
11	Linear	$\sigma_{u\left(j\right)}\sim N\left(\beta_{1}+\beta_{2}G_{j}+\beta_{3}R_{j},\sigma_{r}^{2}\right)$, *σ*_*u(j) *_> 0.0001	-0.16*	0.06*	0.09 (-0.06 0.21)	0.01 (-0.01 0.04)	5601

Parameters marked * have 95% posterior credible intervals which do not include zero. Values quoted are the medians of the posterior distributions. The parameter *β*_1 _is the constant in the equations listed in the third column. The parameter *β*_3 _has been rescaled to millions of inhabitants for clarity.

All models were fitted using WinBUGs [24]. A burn-in period of 50,000 iterations was used during which convergence was completed for all models (as assessed by the Gelman-Rubin statistics and visual analysis of trace-plots of multiple chains). Two chains were then monitored for a further 100,000 iterations from which results were obtained. These lengthy burn-in and sampling periods were required to ensure convergence of the constant *β*_0 _(eqn 2) which was prone to small short-term fluctuations but larger long-term fluctuations. The following priors were assigned: *β*_0-3 _~ dflat(); *σ*_*v *_~ dunif(0,1); model 1 *σ*_*v *_~ dunif(0,1); all other models *σ*_*v *_~ dunif(0,1). Parameter estimates appeared insensitive to alternative prior distributions for *σ*_*v *_or σ_*u*_.

## Results

**Figure 2:**
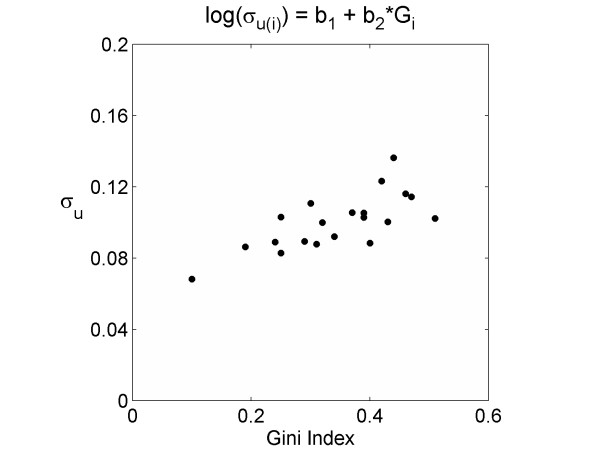
**Between region standard deviations estimated from model 2 against the Gini coefficient for each country**. Between region standard deviations (*σ*_*u(i)*_) which have been estimated from model 2 (Table 2) against the Gini coefficient for each country (Table 1).

**Figure 3:**
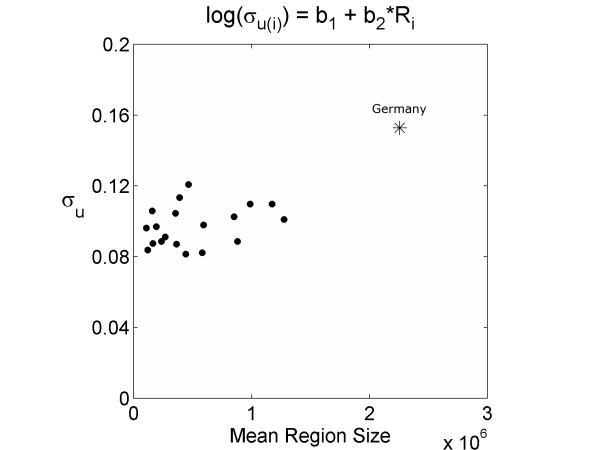
**Between region standard deviations estimated from model 6 against the mean region size for each country**. Between region standard deviations (*σ*_*u(i)*_) which have been estimated from model 6 (Table 2) against the mean region size for each country (Table 1). Germany, with a large mean region size in comparison to the other countries, is represented by a star.

The fitted inter-regional standard deviations at the country level (*σ*_*u*(*j*)_) for the baseline model 1 are given in Table 1. These values represent the estimates of geographical inequalities without taking administrative structure into account. Parameter estimates for the models are presented in Table 2. In all cases, values of *β*_2 _or *β*_3 _that are significantly different from zero indicate that there is a relationship between within-country variation in mortality and either region size inequalities (Gini coefficient) or mean region size respectively. The slope parameters *β*_2 _and *β*_3 _are significantly different from zero in the deterministic forms of the relationship but not when variation is allowed around this relationship (c.f. pairs of models [2-7] and [8,9]). Furthermore, this relationship was positive for all model forms indicating that within-country variation tends to increase with both inequality in region size (Figure 2) and the mean region size in each country (Figure 3). However, the relationship with mean region size is difficult to interpret fairly as Germany's mean region size is approximately 75% larger than the next country and may contribute substantially to the relationship. Model fit was assessed using the DIC [25]: lower values indicate better model fit after taking the number of parameters into account. A reduction of approximately 3 to 4 points was obtained for all models using Gini coefficient and/or mean region size as factors affecting within country variation when compared with the basic random effects model, suggesting a noticeably improved fit. There was however no marked improvement in model fit when these more descriptive models are compared (c.f. models 2-9, Table 2). When compared simultaneously, these factors neither improved model fit, nor achieved significance in the slope parameters (models 10 & 11). Furthermore, these descriptors of population structure were not significantly correlated (two-tailed Pearson correlation using number of regions per country as weights: rho = 0.29, p = 0.22).

It is worth considering how these results affect the interpretation of the models. For the linear models, a change in the Gini coefficient (Δ*G*) or mean region size (Δ*R*) for a given country is associated with an absolute increase in the intra-regional standard deviation (*σ*_*u*(*j*)_) of *β*_2_Δ*G *or *β*_3_Δ*R *respectively. This relationship is complicated for the log models by the exponential function. In these models it is easier to conceptualise how an absolute increase in either population size inequality (*G*) or mean region size (*R*) causes a *proportional *increase in the regional variation. An increase in the Gini coefficient of 0.1 is associated with a 12-14% increase (on average) in regional standard deviation (depending on whether the relationship is deterministic or fitted) in the log models (Table 2, models 2 and 3). Similarly, an increase in mean population size of the regions of 500,000 is associated with a 10% increase (on average) in regional standard deviation (Table 2, models 6 and 7). However, what actually constitutes a low or a high inter-regional level of variation is still somewhat unclear. Country-level variation dominated region-level variation in all the models: *σ*_ν _had a median value of 0.36 (95% credible interval 0.27, 0.52), compared to *σ*_*u*(*j*) _which consistently took an average value of 0.10 in all models. The null model had a range of within country standard deviations from 0.05 to 0.18, whereas the more complex models typically had a range about half this, between approximately 0.07 and 0.14. These within country standard deviations describe the extent of the geographic inequalities between regions within a country that cannot be explained by either mean region size or inequality in region size. To put these values into context, a within-country standard deviation of 0.07 (the country with the lowest degree of inequality between regions) is roughly equivalent to the standardised mortality ratio of a notional geographic unit lying on the 95^th ^centile being 26% higher than that of a region on the 5^th ^centile. For the country with the highest inequalities the standard deviation of 0.14 equates to a 59% excess mortality rate for a region at the 95^th ^centile over a region at the 5^th^.

## Discussion

Several recent epidemiological studies have addressed the issue of variation in geographical units from the point of view of choosing the hierarchical level most appropriate for the observed data. The general finding is that the smaller the geographical unit used, the better the models explain the data with greater clustering of ecological factors [12,16,26,27]. Although the focus of these studies differs, all are concerned with the size of geographic unit. Whereas the previous studies focused on improving the accuracy with which health attributes can be explained by selecting explanatory factors at different spatial scales, we were concerned with whether differences in geographic structure may complicate direct comparison of health inequalities [8].

We used Gini coefficients to describe inequalities in regional population sizes within a country as a measure of geographic structure. Inequalities in regional population size were positively correlated with inter-regional variation (*σ*_*u*(*j*)_) and including this relationship offered an improvement in model fit compared with the random effects model, although this relationship was not significant at conventional statistical levels. Restricting the variation around these relationships to be near-deterministic did yield significant coefficients (as we might expect). One possible motivation for assuming a deterministic relationship would be if there were firm theoretical reasoning to suggest a quantifiable and definite effect of population structure within the hierarchy; we are unaware of any such information in the current literature but suspect that further work in this area may provide clues as to what shape such a relationship may take. However, it would be impossible to generalise our findings to all datasets, whether our models were statistically significant or not, hence there is a need to test these ideas on a case-by-case basis in further datasets at different geographical scales. Indeed further studies including socio-economic factors as explanatory factors would be interesting to see as the patterns we observe may result from underlying causes other than just variations in region size. Undoubtedly international comparisons of socio-economic inequalities in health or mortality would be better assessed using individual data including a harmonised measure of socio-economic position - such as education - where these are available [4,28].

We also found some evidence, but to a lesser degree than for the Gini coefficient, supporting a positive relationship between the mean region size in a country and inter-regional variation, and including this relationship offered a similar improvement in model fit to the Gini coefficient. However, the lack of countries in our dataset with mean region sizes lying between those in Germany (2.3 million) and Italy (1.3 million) makes us wary of drawing a firm conclusion on the validity of the link between health inequalities and mean region size at this scale.

When using ecologic or aggregate data we must avoid committing the ecological fallacy [29,30]; this is closely related to the MAUP discussed earlier in so much as it is a bias caused by the aggregation of individual level data. The fallacy is an error in the interpretation of such data and assumes that relationships found at the group level also hold at the individual level. We therefore stress that, in this study, we are not drawing conclusions about individuals but are comparing average rates and inequalities at the population level. It should also be noted that there are various other methods of comparing health inequalities between countries, or indeed between smaller areas or the same areas at different time points. For example, Mackenbach et al [4] compared health inequalities between socioeconomic groups across 22 European countries using two regression-based measures - the relative index of inequality and the slope of index inequality. Leclerc et al [31] compared inequalities in mortality between England and Wales, France, and Finland using the Gini coeffient as a measure of health inequality. Whereas Leyland et al [32] used various measures to compare inequalities in mortality within Scotland over time, including comparing absolute differences in standardised rates between regions and socioeconomic groups at different time points, comparing socioeconomic rate ratios and examining the slope index of inequality. No matter what method is used to explore such health inequalities one must be aware that the administrative structure of the populations under examination may be influencing their interpretation of variations in health.

## Conclusions

This study suggests that countries or regions comprising unequally sized geographic units may have a tendency to show greater health inequalities simply because of inherent differences in geographic structure. When examining health inequalities between areas it is important to be aware of the potential for such biases and we recommend that when one presents these health inequalities they should also report a simple measure of inequalities in population structure - such as the Gini coefficient - alongside their results. This may be directly applicable to the UK where historical events have led to fundamental differences in the size distribution of administrative regions in the present day, that may themselves exacerbate or occlude existing health inequalities [8]. Furthermore, a move towards smaller more uniformly sized geographic units in the UK during Local Government Reorganisation in Great Britain between 1995 and 1998 [33] may similarly affect accurate description and comparison of geographic health inequalities across this timeline, if there are effects similar to those reported herein. Further work in this area may provide useful information when seeking compromise between statistical, administrative and cultural conflicts over the definition of population boundaries [34]. Whilst it may be possible to achieve consistency in region size within a country either spatially or over time [13,14], it is unlikely that consensus will be achieved between countries and comparisons should be made with care. We expect widespread differences between countries in terms of the geographical units used for reporting data and hence the description of health inequalities may be more affected by the hierarchical structure than considered previously. A better understanding of how differences in geographic structure of a population affect the description and interpretation of health inequalities will improve spatio-temporal monitoring of health inequalities and better inform the evaluation of interventions.

## Competing interests

The authors declare that they have no competing interests.

## Authors' contributions

AL initiated the study. AJ analysed and interpreted the data and wrote the paper. AL and CD assisted with the analysis. AL and CD commented on and revised the paper.

## Funding

The Social and Public Health Sciences Unit is jointly funded by the Medical Research Council and the Chief Scientist Office (CSO) of the Scottish Government Health Directorates (wbs U.1300.00.001.).

## Pre-publication history

The pre-publication history for this paper can be accessed here:

http://www.biomedcentral.com/1471-2288/10/74/prepub
